# β-Caryophyllene Ameliorates Thioacetamide-Induced Liver Fibrosis in Rats: A Preventative Approach

**DOI:** 10.3390/ijms26178493

**Published:** 2025-09-01

**Authors:** Lujain Bader Eddin, Amar Mahgoub, Saeeda Almarzooqi, Ernest Adeghate, Sandeep B. Subramanya, Shreesh Ojha

**Affiliations:** 1 Department of Pharmacology and Therapeutics, College of Medicine and Health Sciences, United Arab Emirates University, Al Ain P.O. Box 15551, United Arab Emirates; 201970113@uaeu.ac.ae (L.B.E.); amar.omer@uaeu.ac.ae (A.M.); 2 Department of Pathology, College of Medicine and Health Sciences, United Arab Emirates University, Al Ain P.O. Box 15551, United Arab Emirates; saeeda.almarzooqi@uaeu.ac.ae; 3 Department of Anatomy, College of Medicine and Health Sciences, United Arab Emirates University, Al Ain P.O. Box 15551, United Arab Emirates; eadeghate@uaeu.ac.ae; 4 Department of Physiology, College of Medicine and Health Sciences, United Arab Emirates University, Al Ain P.O. Box 15551, United Arab Emirates; sandeep.bs@uaeu.ac.ae; 5 Zayed Bin Sultan Center for Health Sciences, United Arab Emirates University, Al Ain P.O. Box 15551, United Arab Emirates

**Keywords:** cannabinoids, liver fibrosis, *Cannabis*, cannabinoid type 2 receptors, prevention, Thioacetamide

## Abstract

Liver fibrosis is associated with increased rates of morbidity and mortality. At present, there are no specific treatments that can directly reverse hepatic fibrosis. The endocannabinoid system has been found to play a significant role in regulating the development and progression of liver diseases, in addition to having protective effects. In this study, we investigate the protective potential of β-Caryophyllene (BCP) against Thioacetamide (TAA)-induced liver fibrosis. Wistar rats were injected with TAA (200 mg/kg) three times per week for 8 weeks to induce liver fibrosis. They also received oral BCP before the TAA injections. AM630 (1 mg/kg) was administered to confirm the CB2 receptor-dependent effect of BCP. The BCP treatment (50 mg/kg) protected against cell injury and potentiated antioxidant defense by replenishing hepatic GSH, improving catalase activity, and inhibiting the formation of MDA. The co-administration of BCP mitigated the TAA-induced inflammatory response by decreasing the release of proinflammatory cytokines. Histological examination showed preserved cellular integrity, decreased collagen deposits with other extracellular matrix proteins, and low levels of myofibroblast activation. In addition, the BCP-treated rats demonstrated upregulated sirtuin 1 (SIRT1) expression, which had a direct inhibitory effect on hypoxia inducible factor (HIF-1α). AM630 pre-treatment inhibited all the aforementioned protective mechanisms of BCP. Based on our findings, BCP exerts protective effects in liver fibrosis, which can be attributed to its agonist action on CB2 receptors. This study provides preclinical evidence of the potential preventative benefits of BCP in liver fibrosis.

## 1. Introduction

Liver fibrosis is a clinical condition that affects patient morbidity and mortality. Fibrosis is a result of hepatocellular damage in response to a variety of inducers. Persistent liver fibrosis eventually leads to liver cirrhosis, which is associated with hepatocellular carcinoma and eventually results in liver failure [1]. Liver fibrosis is induced in response to hepatocyte death, inflammation and hepatic stellate cell (HSC) activation regardless of the etiology, resulting in the accumulation of connective tissue proteins, mainly collagen types I and III, scar formation, and epithelial or endothelial barrier disruption. Damaged hepatocytes release TNF-a, IL-6, and TGF-β, which activate the primary response cells, HSCs, turning them into fibrogenic myofibroblasts that trigger the release of collagen type III. Excessive oxidative stress, which leads to cellular demise and apoptosis, further contributes to the development of liver fibrosis [2]. Chronic liver disease, which includes fibrosis and cirrhosis, is one of the leading causes of mortality worldwide, ranking as the 11th most common cause of death. Non-alcoholic fatty liver disease (NAFLD) and alcoholic liver disease (ALD)-related etiologies are of serious concern, given alcohol consumption rates and the expected increase in obesity [3]. Liver cirrhosis is considered a major health burden because it results in a high number of disability-adjusted life-years and considerable expenditure on hospital-based services for inpatient and emergency care [4]. Liver diseases cause about 2 million deaths each year, comprising 4% of global mortality. Acute cases often stem from viral infections or drug-induced liver injury, while chronic liver disease is mostly due to alcohol use, hepatitis B (HBV), hepatitis C (HCV), and the increasing prevalence of metabolic dysfunction-associated steatotic liver disease (MASLD). These chronic issues can progress to severe outcomes like cirrhosis and liver cancer, significantly impacting global health [5].

Many new therapeutic targets have been explored. One of the most important druggable endogenous targets identified in recent years is the endocannabinoid system (ECS), which comprises endocannabinoids, cannabinoid receptors 1 and 2 (CB1 and CB2, respectively), and enzymes that regulate the turnover of these compounds. Arachidonoyl ethanolamide (AEA, anandamide) was the first endogenous ligand discovered in the ECS, followed by 2-arachidonoyl glycerol (2-AG). Two enzymes are responsible for their degradation: fatty-acid amide hydrolase (FAAH) hydrolyzes AEA and monoglyceride lipase (MAGL) degrades 2-AG [6]. The immunomodulatory effect of the endocannabinoid system (ECS) has sparked significant interest as a potential therapeutic target to regulate the development and progression of liver diseases and provide protective effects [7]. Studies have found CB2 receptors localized in various parts of the body, including cardiomyocytes [8], lungs [9], bone [10], kidneys [11], the liver [12], and the reproductive system [13], indicating their therapeutic potential in various pathological conditions [12,14,15,16,17].

β-caryophyllene (BCP; (E)-BCP) is a lipophilic bicyclic sesquiterpene commonly found in natural essential oils of ornamental and dietary plants, including spices. It is also a constituent of *Cannabis*, with an abundance of up to 35%. BCP is a selective CB2 receptor agonist with a constant inhibition of Ki = 155 ± 4 nM, unlike traditional cannabinoids, which lack strong receptor binding affinity [18]. BCP lacks affinity toward CB1 receptors and is therefore free of unfavorable psychoactive side effects [19,20]. Experimental studies demonstrated an anti-inflammatory effect of BCP, which reduces inflammatory mediators and has a potent antioxidant effect with an antifibrotic effect in many organs [21,22,23,24,25].

Many chemicals can induce liver fibrosis, and Thioacetamide (TAA), an organosulfur compound, has been popularly used to induce liver fibrosis in experimental animals since 1989. When TAA is metabolized to its reactive metabolite, thioacetamide-S-oxide (TASO), it forms reactive adducts with cellular proteins and lipids. This in turn releases an array of reactive intermediates that cause severe oxidative damage to the liver, resulting in cellular destruction and hepatocyte damage. Administration of TAA to experimental rats led to increased production of MDA (malondialdehyde), 4-hydroxynonenal, advanced glycation end products, total nitrite, and nitrate and increased myeloperoxidase activity [26,27,28]. TAA plays a role in triggering an inflammatory response through direct interactions with immune cells. Injured Kupffer cells release inflammatory cytokines and chemokines that facilitate the activation and recruitment of other immune cells to the site of injury [29].

In this study, we aimed to investigate the biological activity of BCP in TAA-induced hepatotoxicity and fibrosis via targeting CB2 receptors and the AMPK/SIRT1/HIF-1α signaling pathway.

## 2. Results

### 2.1. Liver Index

**Figure 1:**
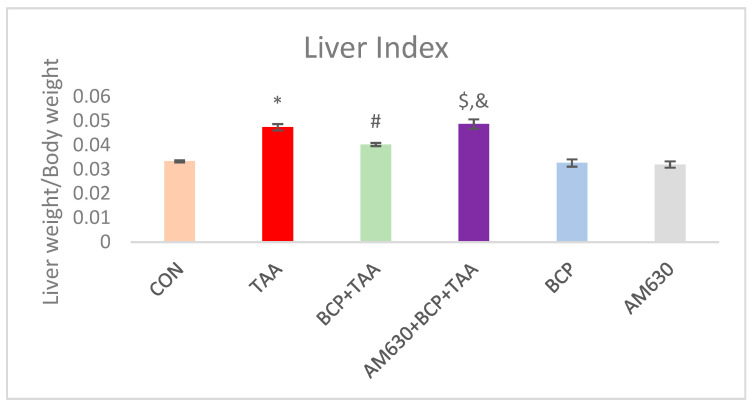
Effect of BCP on TAA-induced change on liver index. * *p* < 0.05 CON vs. TAA, ^#^ *p* < 0.05 TAA vs. BCP + TAA, ^$^ *p* < 0.05 BCP + TAA vs. AM630 + BCP + TAA, ^&^ *p* > 0.05 (n.s.) AM630 + BCP + TAA vs. TAA (one-way ANOVA followed by DMRT). CON: Control, TAA: Thioacetamide, BCP: β-Caryophyllene, BCP + TAA: β-Caryophyllene + Thioacetamide treatment, AM630 + BCP + TAA: β-Caryophyllene + AM630 + Thioacetamide.

After eight weeks of administration, TAA caused a significant increase in the liver index, which reflects a decreased body weight and increased liver mass compared to a CON group. In contrast, BCP treatment restored a relatively normal liver index in rats by enabling maintenance of normal body weight and prevention of increased liver weight. On the contrary, a group that received AM630 + BCP + TAA exhibited an elevated liver index similar to the TAA group, which can be attributed to the counteracting effect of AM630 on BCP (Figure 1).

### 2.2. Effect of BCP on Serum Parameters

**Figure 2:**
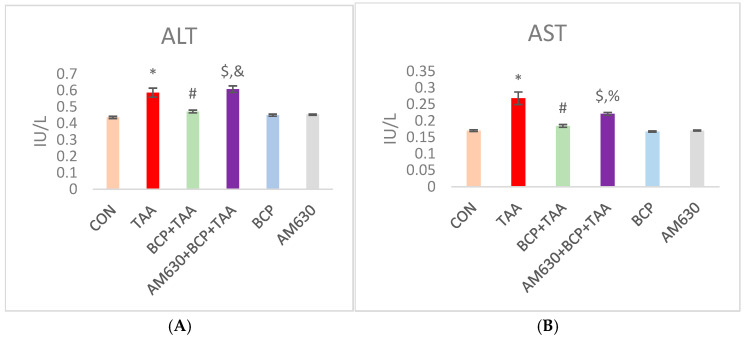
Effect of BCP on TAA-induced liver damage. (**A**) ALT level, (**B**) AST level. * *p* < 0.05 CON vs. TAA, ^#^ *p* < 0.05 TAA vs. BCP + TAA, ^$^ *p* < 0.05 BCP + TAA vs. AM630 + BCP + TAA, ^%^ *p* < 0.05 AM630 + BCP + TAA vs. TAA, ^&^ *p* > 0.05 (n.s.) AM630 + BCP + TAA vs. TAA, (one-way ANOVA followed by DMRT). CON: Control, TAA: Thioacetamide, BCP: β-Caryophyllene, BCP + TAA: β-Caryophyllene + Thioacetamide treatment, AM630 + BCP + TAA: β-Caryophyllene + AM630 + Thioacetamide.

Both ALT and AST are crucial injury markers for liver function. As shown in Figure 2, the levels of ALT and AST were significantly elevated in the rats with TAA-induced liver damage compared to the CON rats (*p* < 0.05). Administration of BCP led to a significant reduction in ALT and AST levels compared to the group that received TAA alone. In addition, the concomitant administration of AM630 with BCP inhibited BCP’s mediating effect, as indicated by the increased levels of ALT and AST in the AM630 + BCP + TAA group compared to the BCP + TAA group. Both the BCP and AM630 alone groups showed no significant change in functional parameters.

### 2.3. Effect of BCP on Oxidative Stress

**Figure 3:**
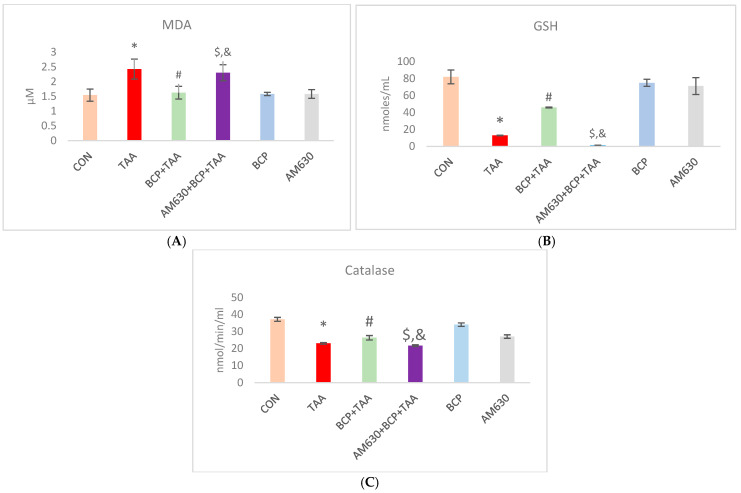
Effect of BCP treatment on TAA-induced oxidative stress. (**A**) MDA concentration, (**B**) GSH content, and (**C**) catalase activity. Data is presented as mean ± SEM (*n* = 9). * *p* < 0.05 CON vs. TAA, ^#^ *p* < 0.05 TAA vs. BCP + TAA, ^$^ *p* < 0.05 BCP + TAA vs. AM630 + BCP + TAA, ^&^ *p* > 0.05 (n.s.) AM630 + BCP + TAA vs. TAA (one-way ANOVA followed by DMRT). CON: Control, TAA: Thioacetamide, BCP: β-Caryophyllene, BCP + TAA: β-Caryophyllene + Thioacetamide treatment, AM630 + BCP + TAA: β-Caryophyllene + AM630 + Thioacetamide.

Hepatic oxidative status was evaluated by assessing MDA (a lipid peroxidation byproduct) and GSH (a tripeptide antioxidant) concentration and catalase enzyme activity. TAA administration induced oxidative stress, as indicated by a significant increase in MDA concentration compared to the CON group (Figure 3A). At the same time, TAA drastically reduced the GSH concentration and decreased catalase activity (Figure 3B,C). Treating TAA-injected rats with BCP improved their oxidative status, as indicated by the reduced MDA concentration, replenished GSH content, and increased catalase activity compared to the TAA-injected rats. In addition, the administration of AM630 in the AM630 + BCP + TAA group reversed the protective effect of BCP observed in the BCP + TAA group. The AM630 + BCP + TAA-treated group exhibited a significant increase in oxidative stress, as indicated by an increased MDA concentration, reduced GSH concentration, and decreased catalase activity. BCP or AM630 alone did not induce any significant changes in the oxidative markers compared with the CON group.

### 2.4. Effect of BCP on Inflammation

**Figure 4:**
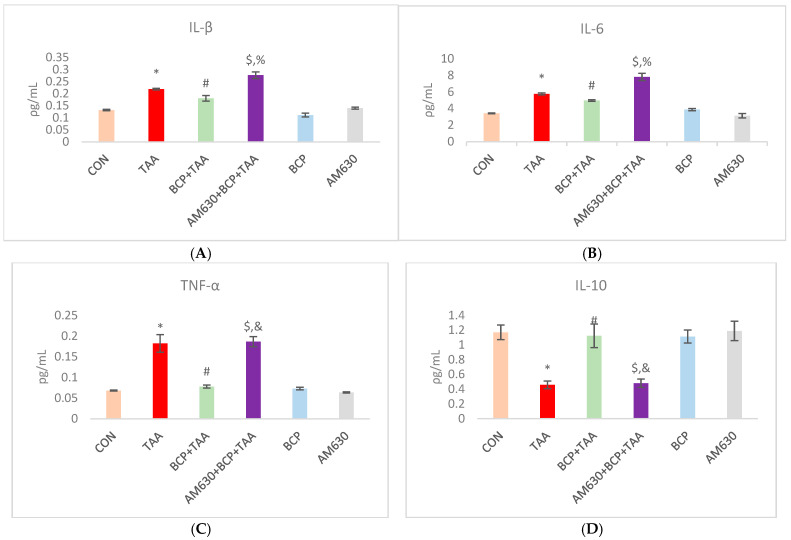
Effect of BCP treatment on inflammatory cytokine expression in livers of TAA-treated rats. (**A**) Interleukin-1β (IL-1β), (**B**) interleukin-6 (IL-6), (**C**) tumor necrosis factor-α (TNF-α), and (**D**) interleukin-10 (IL-10) levels were measured in rat livers. Data is presented as mean ± SEM (*n* = 8). * *p* < 0.05 CON vs. TAA, ^#^ *p* < 0.05 TAA vs. BCP + TAA, ^$^ *p* < 0.05 BCP + TAA vs. AM630 + BCP + TAA, ^%^ *p* < 0.05 AM630 + BCP + TAA vs. TAA, ^&^ *p* > 0.05 (n.s.) AM630 + BCP + TAA vs. TAA (one-way ANOVA followed by DMRT). CON: Control, TAA: Thioacetamide, BCP: β-Caryophyllene, BCP + TAA: β-Caryophyllene + Thioacetamide treatment, AM630 + BCP + TAA: β-Caryophyllene + AM630 + Thioacetamide.

The levels of IL-1β, IL-6, and IL-10 were determined in rat hepatic tissue. The total IL-1β, IL-6, and TNF-α levels were notably elevated in the TAA group compared to the CON group. BCP treatment reduced the levels of IL-1β, IL-6, and TNF-α in the BCP + TAA group compared to the TAA group. Administration of AM630 in the AM630 + BCP + TAA group reverted the levels of inflammatory cytokines to levels similar to those of the TAA only group. In contrast, in the TAA group, the level of IL-10 was significantly decreased when compared to the CON level. In the BCP + TAA group, the IL-10 level was significantly higher than in the TAA group. The AM630 + BCP + TAA group showed a significantly reduced level of the anti-inflammatory cytokine IL-10 compared with the BCP + TAA group (Figure 4A–D).

### 2.5. Histological Examination and Gross Morphology

**Figure 5:**
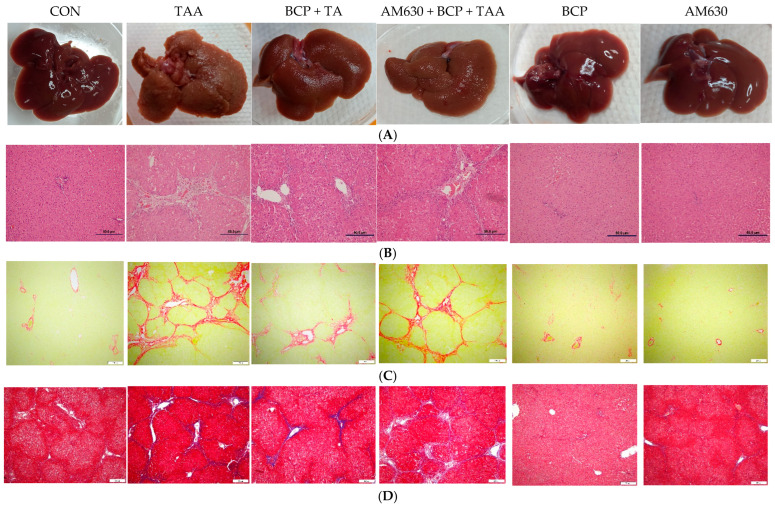
Gross morphology and microscopic images of liver sections. (**A**) Rat livers, (**B**) H&E staining (scale bar is 50 µm), (**C**) Sirius Red staining (scale bar is 200 µm), and (**D**) Masson’s Trichrome staining (scale bar is 200 µm). CON: Control, TAA: Thioacetamide, BCP: β-Caryophyllene, BCP + TAA: β-Caryophyllene + Thioacetamide treatment, AM630 + BCP + TAA: β-Caryophyllene + AM630 + Thioacetamide.

To evaluate the effect of TAA on liver tissue integrity, histological analysis was performed. The CON group showed no induced changes in liver gross morphology (Figure 5A) or damage to the gross architecture, with a clear, smooth liver surface and no nodules. However, the TAA group showed a significant change in gross morphology with the appearance of nodules, shifting the liver surface to coarse in texture and irregular in shape. The BCP + TAA group shows attenuated damage with decreased nodules, similar to the appearance of the CON group livers. The AM630 + BCP + TAA image shows a significant gross morphological change, with obvious induced fibrosis and a nodular appearance. Neither the BCP nor AM630 alone group showed noticeable changes or damage.

Hematoxylin and eosin (H&E) staining of liver sections showed normal lobule architecture and intact cellular shape and hepatocyte organization in the CON group (Figure 5B). The TAA group liver sections showed significant hepatic lobule disarrangement with apparent cellular necrosis and fibrous expansion of portal areas with portal bridging. Liver sections from the BCP + TAA group showed ameliorated induced fibrosis with reserved cellular architecture. The AM630 + BCP + TAA group showed clear resultant fibrosis with inflamed and necrotic cells. Findings for both the BCP and AM630 alone groups were similar the CON group findings.

Sirius Red-stained liver sections from the TAA group indicate a significant deposition of collagen, reflected by an increased area of positive staining compared with the CON group liver sections. Collagen deposition was significantly reduced by the BCP treatment in the BCP + TAA group, as evidenced by decrease in the stained area. In contrast, the combined administration of AM630 + BCP + TAA resulted in liver sections with increased collagen deposition compared to the BCP + TAA group (Figure 5C). BCP and AM630 alone caused no increase in collagen deposition.

The level of liver fibrosis was further determined using Masson’s Trichrome staining. The liver sections from the CON group did not show any signs of collagen accumulation. The TAA group’s sections revealed cirrhosis, with a pattern of hepatic fibrosis and collagen deposition around central vein, portal tracts, and bridging fibrosis. Attenuation of collagen deposition was observed in the BCP + TAA group compared to the TAA alone group. However, antagonizing CB2 receptors in the AM630 + BCP + TAA group resulted in highly increased collagen deposition compared to the BCP + TAA group, reflecting a pattern of cirrhosis (Figure 5D). Neither the BCP nor AM630 alone group showed induced collagen deposition.

### 2.6. Immunohistochemical Staining of Liver Sections

**Figure 6:**
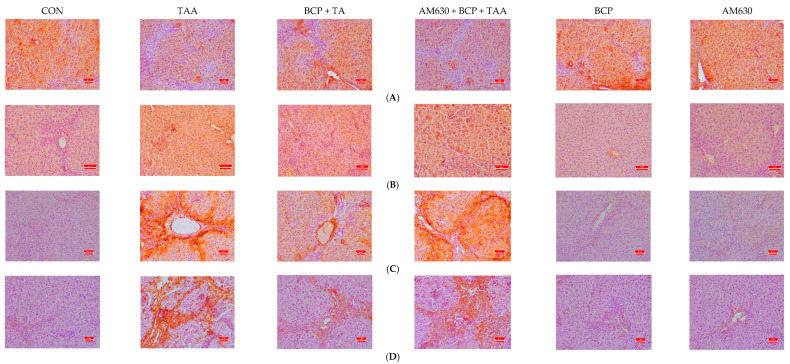

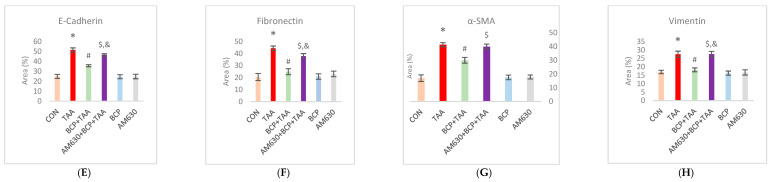
Immunohistochemical staining of liver sections. (**A**) E-Cadherin, (**B**) Fibronectin, (**C**) α-SMA, and (**D**) Vimentin; (**E**–**H**) Quantification of E-Cadherin, Fibronectin, α-SMA, and Vimentin, respectively, (scale bar is 2 µm). Data is presented as mean ± SEM (*n* = 5). * *p* < 0.05 CON vs. TAA, ^#^ *p* < 0.05 TAA vs. BCP + TAA, ^$^ *p* < 0.05 BCP + TAA vs. AM630 + BCP + TAA, ^&^ *p* > 0.05 (n.s.) AM630 + BCP + TAA vs. TAA (one-way ANOVA followed by DMRT). CON: Control, TAA: Thioacetamide, BCP: β-Caryophyllene, BCP + TAA: β-Caryophyllene + Thioacetamide treatment, AM630 + BCP + TAA: β-Caryophyllene + AM630 + Thioacetamide.

The expression of α-SMA, fibronectin, E-cadherin, and vimentin was evaluated in the liver sections. Immunostaining of E-cadherin showed significantly decreased expression in the TAA group compared to the CON group, whereas the BCP-treated group showed restored E-cadherin expression. In contrast, the combined administration of AM630 + BCP + TAA resulted in a decrease in E-cadherin expression compared to the administration of BCP alone (Figure 6A). Fibronectin was highly expressed in the TAA group compared to the CON group. BCP decreased fibronectin expression in the BCP + TAA group compared to the TAA group, whereas the AM630 + BCP + TAA group displayed a high level of fibronectin expression compared to the BCP + TAA group (Figure 6B).

Similarly, in the CON group, the liver sections showed decreased α-SMA staining, indicating less fibroblast activation (Figure 6C). In contrast, the TAA group sections demonstrated a considerable increase in α-SMA expression, indicating an increase in fibroblast activation and fibrosis induction. Rats treated with BCP (BCP + TAA) showed reduced α-SMA expression compared with the TAA-only group. However, α-SMA expression was significantly induced in the AM630 + BCP + TAA group compared with the BCP + TAA group.

Regarding vimentin (Figure 6D), TAA induction strongly increased vimentin expression compared to the CON group. However, vimentin was notably reduced in the BCP-treated group compared to the group that received TAA alone. The combined administration of AM630 and BCP induced upregulation in the expression of vimentin compared to the BCP + TAA group. The expression levels of E-cadherin and vimentin observed following TAA administration indicate an increase in the transition of endothelial cells to mesenchymal cells.

### 2.7. Effect of BCP on FGF-18 and α-SMA Expression

**Figure 7:**
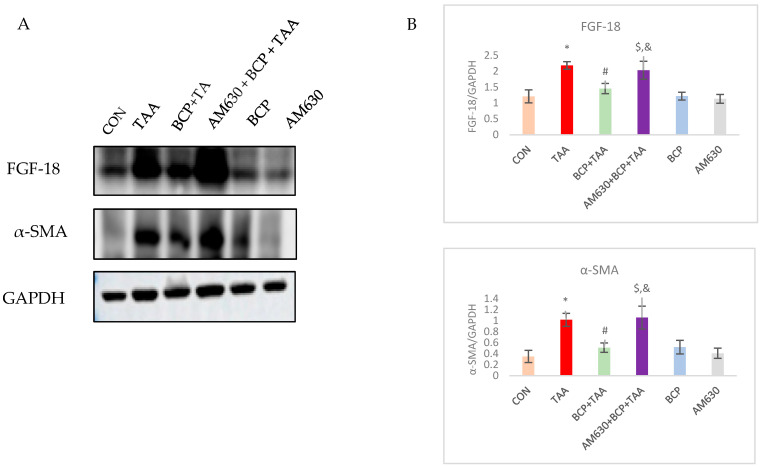
Expression of FGF-18 and α-SMA in liver tissue. (**A**) Western immunoblots of FGF-18 and α-SMA. (**B**) Corresponding densitometric quantification of their expression. Data is presented as mean ± SEM (*n* = 3). * *p* < 0.05 CON vs. TAA, ^#^ *p* < 0.05 TAA vs. BCP + TAA, ^$^ *p* < 0.05 BCP + TAA vs. AM630 + BCP + TAA, ^&^ *p* > 0.05 (n.s.) AM630 + BCP + TAA vs. TAA (one-way ANOVA followed by DMRT). CON: Control, TAA: Thioacetamide, BCP: β-Caryophyllene, BCP + TAA: β-Caryophyllene + Thioacetamide treatment, AM630 + BCP + TAA: β-Caryophyllene + AM630 + Thioacetamide.

FGF-18 is a growth factor that promotes the proliferation and activation of fibroblasts. FGF-18 was highly upregulated in the TAA group compared to the CON group. However, there was a notable decline in its expression in the rats that received BCP with their TAA injections. The administration of AM630 led to an upsurge in FGF-18 expression through inhibiting BCP-mediated CB2 receptor activation. To evaluate the activation of fibroblasts, we studied α-SMA expression among the groups. Immunoblotting showed upregulated α-SMA expression in the TAA-treated rats compared to the CON group. The rats treated with BCP showed lower α-SMA expression compared with the TAA-only group. Consistent with the TAA group, the AM630 + BCP + TAA group exhibited increased α-SMA expression when compared to the BCP + TAA group (Figure 7).

### 2.8. Effect of BCP on Expression of AMPK-SIRT1-HIF1

**Figure 8:**
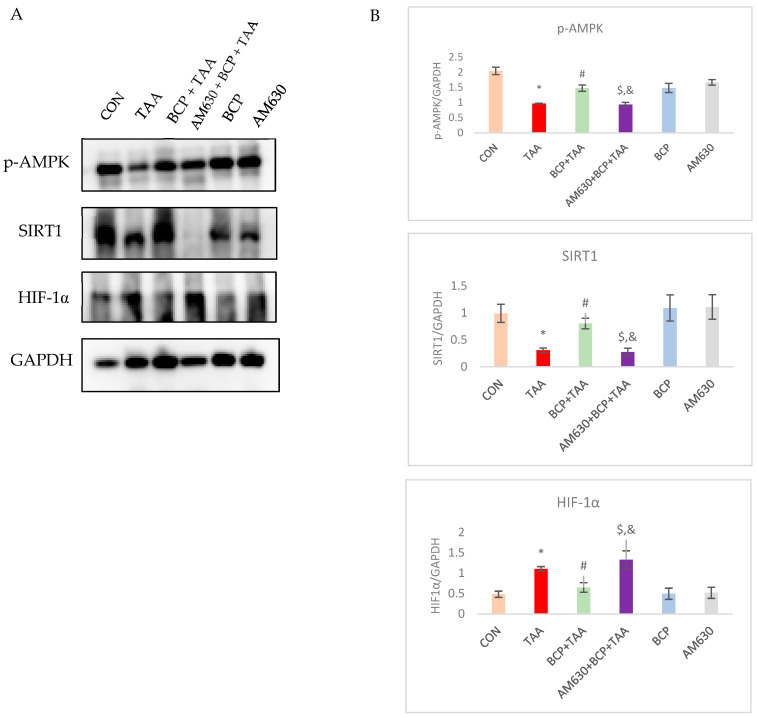
Expression of p-AMPK, SIRT1, and HIF1-α in liver tissue. (**A**) Western blot analysis of p-AMPK, SIRT1 and HIF1-α. (**B**) Qualification of immunoblots. Data is presented as mean ± SEM (*n* = 3). * *p* < 0.05 CON vs. TAA, ^#^ *p* < 0.05 TAA vs. BCP + TAA, ^$^ *p* < 0.05 BCP + TAA vs. AM630 + BCP + TAA, ^&^ *p* > 0.05 (n.s.) AM630 + BCP + TAA vs. TAA (one-way ANOVA followed by DMRT). CON: Control, TAA: Thioacetamide, BCP: β-Caryophyllene, BCP + TAA: β-Caryophyllene + Thioacetamide treatment, AM630 + BCP + TAA: β-Caryophyllene + AM630 + Thioacetamide.

To validate the possible mechanism of BCP’s protective effects against TAA-induced fibrosis, we evaluated the activation of the epigenetic regulator SIRT1. Western blotting analysis showed a clear depletion (*p* < 0.05) of SIRT1 in the TAA group compared to the CON group. Administration of BCP significantly (*p* < 0.05) reinstated the expression of SIRT1 in the TAA-injected rats compared to the TAA-only group. Concomitant administration of AM630 inhibited the BCP-mediated effect, resulting in decreased levels of SIRT1 in the AM630 + BCP + TAA group. Downregulated SIRT1 expression in the TAA group was also correlated with decreased activation of AMPK, which was reversed by the BCP treatment. Immunoblotting showed that TAA induction significantly increased the expression of HIF1-α compared to the vehicle-treated group, which decreased following BCP treatment. In the AM630 + BCP + TAA group, HIF1-α was increased again, which demonstrates the inhibitory effect of AM630 on BCP’s action (Figure 8).

### 2.9. Effect of BCP on TGF-β Signaling Pathway Activation

**Figure 9:**
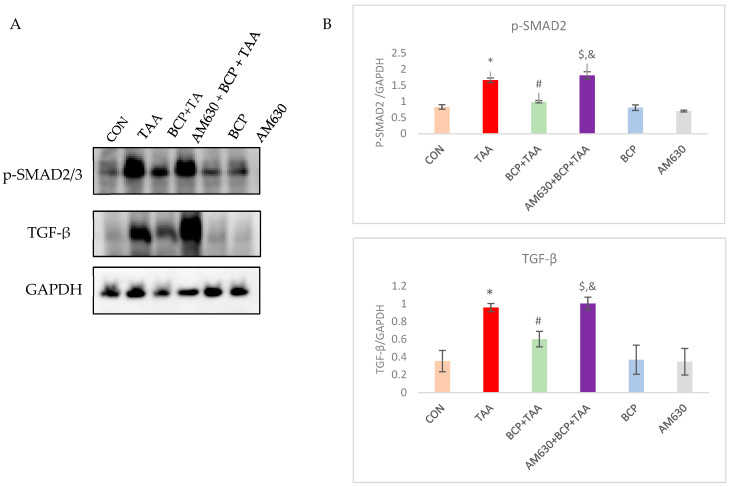
Expression of p-SMAD2 and TGF-β in liver tissue. (**A**) Western immunoblots and the (**B**) corresponding densitometric quantification of p-SMAD2 and TGF-β. Data is presented as mean ± SEM (*n* = 3). * *p* < 0.05 CON vs. TAA; ^#^ *p* < 0.05 TAA vs. BCP + TAA; ^$^ *p* < 0.05 BCP + TAA vs. AM630 + BCP + TAA; ^&^ *p* > 0.05 (n.s.) AM630 + BCP + TAA vs. TAA (one-way ANOVA followed by DMRT). CON: Control; TAA: Thioacetamide; BCP: β-Caryophyllene; BCP + TAA: β-Caryophyllene + Thioacetamide treatment; AM630 + BCP + TAA: β-Caryophyllene + AM630 + Thioacetamide.

TGF-β is a master regulator in fibrogenesis. In this study, we assessed the expression of TGF-β in response to TAA administration and the subsequent activation of its downregulators. TGF-β was strongly induced in the TAA-treated group; however, BCP treatment inhibited its expression. In the AM630 + BCP + TAA group, TGF-β increased sharply compared to its expression in the BCP + TAA group. p-SMAD2 exhibited behavior consistent with TGF-β expression. In response to TAA injection and following increased TGF-β expression, SMAD2 was significantly activated in the TAA group compared to the CON rats. In contrast, SMAD2 phosphorylation was decreased following the concomitant administration of BCP with TAA. In the AM630 + BCP + TAA group, AM630 injection caused an increase in the expression of phosphorylated SMAD2 compared with the BCP + TAA treatment (Figure 9).

### 2.10. Effect of BCP on Matrix Metalloprotease Activation

**Figure 10:**
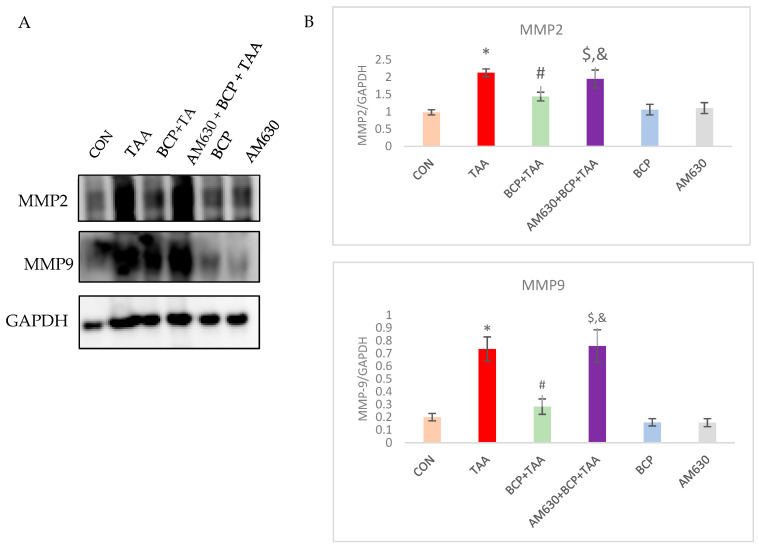
Expression of MMP2 and MMP9 in liver tissue. (**A**) Western immunoblots of MMP2 and MMP9; (**B**) the corresponding densitometric quantification. Data is presented as mean ± SEM (*n* = 3). * *p* < 0.05 CON vs. TAA, ^#^ *p* < 0.05 TAA vs. BCP + TAA, ^$^ *p* < 0.05 BCP + TAA vs. AM630 + BCP + TAA, ^&^ *p* > 0.05 (n.s.) AM630 + BCP + TAA vs. TAA (one-way ANOVA followed by DMRT). CON: Control, TAA: Thioacetamide, BCP: β-Caryophyllene, BCP + TAA: β-Caryophyllene + Thioacetamide treatment, AM630 + BCP + TAA: β-Caryophyllene + AM630 + Thioacetamide.

MMP2 and MMP9 are gelatinases that help degrade collagen. They are essential markers of liver fibrosis progression. Western blot analysis revealed that the expression of both MMP2 and MMP9 was significantly upregulated in the TAA disease control group compared to the CON group. In contrast, BCP pre-treatment resulted in reduced expression compared with the TAA group. On the contrary, the administration of the CB2 receptor antagonist counteracted BCP’s effects and led to notably higher expression of MMP2 and MMP9 in the AM630 + BCP + TAA group compared to the BCP + TAA group (Figure 10).

### 2.11. Effect of BCP on Collagen Expression

**Figure 11:**
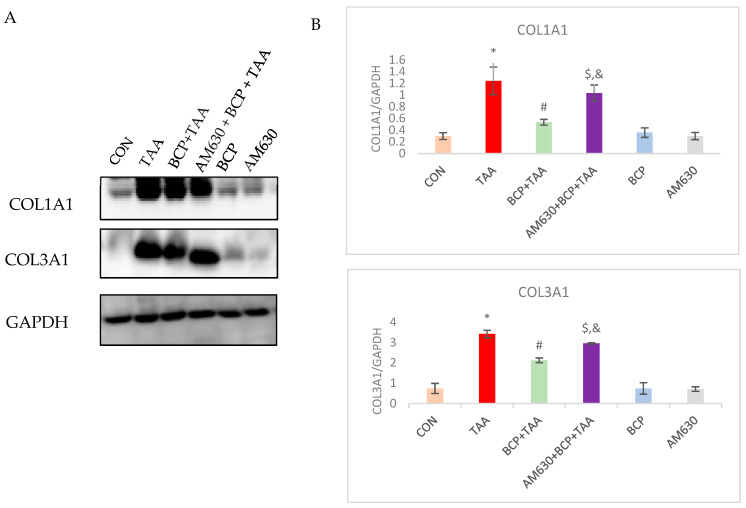
Expression of COL1A1 and COL3A1 in liver tissue. (**A**) Blots of COL1A1 and COL3A1 expression; (**B**) corresponding densitometric quantification of COL1A1 and COL3A1. Data is presented as mean ± SEM (*n* = 3). * *p* < 0.05 CON vs. TAA, ^#^ *p* < 0.05 TAA vs. BCP + TAA, ^$^ *p* < 0.05 BCP + TAA vs. AM630 + BCP + TAA, ^&^ *p* > 0.05 (n.s.) AM630 + BCP + TAA vs. TAA (one-way ANOVA followed by DMRT). CON: Control, TAA: Thioacetamide, BCP: β-Caryophyllene, BCP + TAA: β-Caryophyllene + Thioacetamide treatment, AM630 + BCP + TAA: β-Caryophyllene + AM630 + Thioacetamide.

In order to evaluate the antifibrotic effect of BCP, the expression of different extracellular proteins was assessed. The expression of COL1A1 and COL3A1 was significantly increased (*p* < 0.05) in the TAA group compared to the CON group, whereas the BCP treatment significantly reduced their expression in the BCP + TAA group. The combined treatment (AM630 + BCP + TAA) strongly induced fibrotic marker expression compared to the BCP + TAA treatment. The BCP-only and AM630 groups showed no significant change in fibrotic protein expression (Figure 11).

## 3. Discussion

Liver fibrosis is associated with a decreased survival rate once it progresses to the decompensated phase. It begins with parenchymal injury, which is preceded by a persistent inflammatory response and eliminated by fibrogenesis as part of the wound-healing response to combat necrotic tissue [30]. As there is no clear therapeutic approach to halting the development of liver fibrosis and its associated complications, we investigated the potential protective effect of β-Caryophyllene (BCP) in curbing liver fibrosis. To this aim, we used an experimental model of thioacetamide (TAA)-induced liver fibrosis, modulating the activity of AMP-activated protein kinase (AMPK)/Sirtuin-1 (SIRT-1) and inhibiting hypoxia-inducible factor 1-alpha (HIF-1α). In animal models, TAA is a well-established inducer of hepatic fibrosis that resembles human fibrosis, with an ability to confer irreversible fibrosis (i.e., cirrhosis—end stage liver damage) if administered persistently [31]. In this study, the administration of TAA triggered clear increases in serum ALT and AST, which imply obvious hepatocyte damage and liver dysfunction. The TAA-intoxicated animals experienced hepatomegaly, as demonstrated by their liver/body weight ratio in comparison with CON rats. Our results show that this ratio was maintained following BCP treatment, which indicates that BCP has a preventive effect on the progression of liver fibrosis. Body weight was also observed to be affected after TAA administration. The CON group rats followed a normal growth trend, showing consistent weight gain. In contrast, the rats injected with TAA exhibited weight loss, with an increased liver–body weight ratio. This observation has been reported in other studies [32]. Various studies showed that BCP can decrease the liver index when compared to a diseased control group [23,25].

We also noted an evident increase in oxidative stress, with an increase in malondialdehyde (MDA) and simultaneous declines in the hepatic level of glutathione (GSH) and catalase activity following TAA administration. The oxidative injury induced by TAA is attributed to its reactive metabolites, which promote the production of reactive oxygen species (ROS). This increased ROS production is associated with impaired intracellular glutathione (GSH) homeostasis. GSH is involved in the body’s antioxidant defense by protecting cellular macromolecules from oxidizing reactive species [33]. BCP treatment reduced the hepatic level of MDA and enhanced endogenous antioxidant defense, as represented by a potentiated GSH level and catalase activity. An antioxidant effect of BCP was also reported through its inhibition of ROS production and NADPH oxidase (NOX) 2/4 activity [34].

Unresolved inflammation is also implicated in liver fibrosis induction, in which levels of various inflammatory mediators are notably increased [35]. In this study, we demonstrated the anti-inflammatory activity of BCP against TAA-induced fibrosis. Following BCP treatment, the inflammatory markers IL-1β, IL-6, and TNF-α decreased considerably concomitantly with an increase in the release of the anti-inflammatory cytokine IL-10. The protective effect of BCP has been extensively demonstrated in disorders associated with chronic inflammation [36]. Those findings support the anti-inflammatory effects of BCP in the liver and show it to mitigate liver fibrosis by inhibiting the activation of macrophages and related inflammatory molecules.

Both sustained oxidative stress and inflammation lead to fibrosis, which manifests as accumulated extracellular matrix proteins, including collagen types I and III and fibronectin. At the same time, hepatic stellate cells are activated into myofibroblasts with enhanced expression of α-SMA, which a commonly used as a biomarker for myofibroblast activation [37,38]. In the course of resolving the inflammatory and pro-fibrotic phase, ECM turnover becomes dysregulated, resulting in altered collagen deposition, forming a collagenous scar and leading to fibrosis. Therefore, we further investigated the effect of BCP on fibrotic markers in TAA-induced fibrosis. It is well-documented that TAA administration is associated with upregulated expression of fibrotic markers and increased activation of myofibroblasts [39,40]. Our results evidence the effects of BCP in hepatic fibrosis via reduced fibronectin expression. BCP also attenuated the concomitant activation of myofibroblasts, as shown by the decrease in α-SMA expression. Our histological examination demonstrates the protective potential of BCP against TAA-induced liver damage. In the TAA group, liver sections revealed significant structural damage, with clear micronodules evident in the gross morphology of the liver, along with irregular lobules and necrotic cells. Detectable accumulated collagen was observed in the TAA group, whose livers showed dense fibrous septa and condensed central veins. BCP treatment decreased collagen accumulation while retaining a considerable amount of deposited collagen, as evidenced by Masson’s trichrome and Sirius Red staining, along with intact nuclei and cellular structural integrity, as shown via H&E staining. These results concur with other phytochemical studies, which have shown protective effects against TAA-induced fibrosis [41,42]. MMPs are proteolytic enzymes that degrade ECM components, and their activity is inhibited by tissue inhibitor of metalloproteinases (TIMP). During the fibrogenic response, ECM proteins build up, and MMPs are released to facilitate the required degradation. However, activated hepatic stellate cells release TIMPs, thus inhibiting MMPs, which leads to further accumulation of proteins and subsequent release of MMPs, repeating the cycle [43]. In this study, we observed a significant elevation in the protein expression levels of MMP2 and MMP9 following TAA injection. However, the co-administration of BCP significantly downregulated their expression compared with the TAA group.

The endothelial–mesenchymal transition (EMT), in which epithelial cells lose their polarity and transform into interstitial cells, is involved in mediating fibrogenesis. Hepatocytes, bile duct cells, and hepatic stellate cells can be transformed into myofibroblasts through the EMT [44]. In our study, vimentin levels were significantly increased while E-cadherin expression was reduced in the TAA-treated rats; however, BCP treatment mitigated the TAA-induced EMT. Fibroblast growth factors (FGFs) are involved in cellular repair, proliferation, and wound healing in different organs. FGFs promote cellular regeneration in the liver upon injury. However, overexpressed FGFs facilitate hepatocellular carcinoma development following liver fibrosis [45]. FGF-18 is a member of the FGF family that is known for its growth-promoting effect on hepatocytes [46]. It is also recognized for its increased expression in liver fibrosis, which it promotes [47]. Our results revealed a considerable increase in the expression of FGF-18 in the TAA group. In contrast, BCP treatment reduced FGF-18 expression to a relatively normal level. Another master regulator of fibrogenesis is TGF-β, which has a prominent role in inducing the activation of hepatic stellate cells, recruiting inflammatory cells, and stimulating the release of various fibrotic markers [48]. TAA administration is corroborated with high levels of expression of TGF-β, which in turn acts through the SMAD-dependent pathway to cause the phosphorylation of SMAD2 and SMAD3 and increase the transcription of fibrotic markers. Treating the TAA-injected rats with BCP resulted in a significant decrease in the expression of both TGF-β and phosphorylated SMAD2.

Sirtuin 1 (SIRT1) is a nicotinamide adenine dinucleotide (NAD+)-dependent deacetylase that removes the acetyl group of the target protein. SIRT1 is involved in a wide range of cellular physiological functions. Its functions extend to alleviating inflammation and oxidative stress and therefore attenuating associated fibrosis. AMPK is a phosphorylating kinase that modulates the activation of SIRT1 by raising intracellular NAD+ and phosphorylating SIRT1 at Ser27. It is reported that the level of SIRT1 transcription is decreased in non-alcoholic fatty liver disease (NAFLD) patients compared with normal individuals [49]. Experimental NAFLD animals also showed decreased SIRT1 expression following fibrosis induction with various inducers [50,51]. TAA administration is known to induce liver fibrosis through the ablation of SIRT1 activity, which is associated with deactivated AMPK [52]. Extensive crosstalk between SIRT1 and oxidative stress has been reported. ROS accumulation in mitochondria with decreased ATP production and increased AMP concentration impair AMPK function [53]. Activation of AMPK is correlated with suppressed hepatic stellate cell activation [54]. Taking into consideration the regulatory effect of SIRT1 on oxidative stress, mitochondrial stability, apoptosis, and inflammation, decreased AMPK function reduces SIRT1 function and therefore exacerbates both inflammatory and oxidative responses in hepatocytes. The protective results of BCP in this study can be explained in light of BCP’s ability to reverse the reduction in the AMPK/SIRT1 axis. Pharmacological activation of SIRT1 by BCP has been reported, which implies the therapeutic potential of BCP in treating different disease complications [55]. This study showed that TAA has an effect on induceing HIF1-α, which is corroborated by a previous study that found TAA to induce HIF1-α elevation [56]. HIF1-α is implicated in liver fibrosis, in which it augments oxidative injury; chronic HIF1-α expression was found to mediate apoptosis and liver injury [57,58]. Interestingly, BCP administration was shown to reinstate TAA-induced HIF1 α and therefore attenuate resulting oxidative damage by enhancing the function of SIRT1 through resorting AMPK activity, as has been previously documented with other compounds [55,59]. Accordingly, our findings reveal the protective role BCP exerts through modulating AMPK/SIRT1/HIF1-α and thus protecting against liver fibrosis. As shown by all investigated parameters, the pre-administration of AM630, a selective CB2 receptor antagonist, inhibited BCP-mediated protective effects, confirming that mechanisms of BCP’s effects are CB2-receptor dependent and achieved through the activation of CB2 receptors expressed on Kupffer cells and hepatocytes.

This study was exclusively conducted on male rats to minimize the hormonal variability associated with the estrous cycle in female rats, which is associated with significant fluctuations in estrogen, a hormone known to play a role in liver fibrosis development and progression. As such, the exclusion of female rats was intended to provide a consistent and established baseline model for liver fibrosis that excludes confounding factors, such as the hormonal variability observed in females. Given the fact that hormonal influence is temporary and eliminated with menopause, at which point the risk of liver fibrosis becomes equivalent to the risk for males, investigations in a stable and standard sample are necessary [60].

Epidemiological studies have also shown an increased prevalence and incidence rate of liver fibrosis in men compared to women. Given the protective effect of estrogen against fibrosis and the subsequent slow development of fibrosis in females, we favored males in order to reach the maximum severity in the assigned duration [61,62,63,64].

This study’s approach was also based on a precedent set by the literature, in which many foundational studies have been conducted on male rodents. Therefore, building upon these findings helps maintain continuity and comparability with reported data [65,66]. These studies, along with many others, are well-cited and used extensively as a basis for carrying out mechanistic and therapeutic investigations. Moreover, most studies conducted on female rodents aimed to specifically investigate the effect of estrogen on fibrosis and its development and did not generate a standardized model [67,68,69]. Although the use of male rats is justified and aligns with previous studies, the exclusion of female rats limits generalizability and remains a limitation. The inclusion of both sexes allows for broader applicability and enhances translational relevance, providing a comprehensive understanding of the investigated effect. Therefore, incorporating female subjects would offer valuable insights into potential sex-specific responses and improve the robustness of our findings.

## 4. Materials and Methods

### 4.1. Drugs and Chemicals

Thioacetamide and β-Caryophyllene were purchased from Thermo Fisher Scientific (Ward Hill, MA, USA) and Sigma-Aldrich (St. Louis, MO, USA), respectively. AM630 was procured from GLPBIO (Montclair, CA, USA, Cat. No. GC10147). All compounds were prepared fresh daily before treatment administration. Thioacetamide was dissolved in distilled water, and β-Caryophyllene (BCP) was prepared in scientific-grade light olive oil. AM630 was dissolved in 2 mL of dimethyl sulfoxide (DMSO) with 200 µL of Tween-80 and finally prepared in normal saline.

### 4.2. Experimental Animals

Male albino Wistar rats aged six weeks (180–240 g) were sourced from the animal research facility at the College of Medicine and Health Sciences, United Arab Emirates University. The animals were housed in standard conditions at the animal research facility and allowed unlimited food and water, with a day and night cycle with an interval of 12 h. An acclimatization period of one week preceded the start of the experiment. The experiment was performed in accordance with animal ethics guidelines and approved by the animal ethics committee of United Arab Emirates University (No. ERA_2025_5826).

### 4.3. Experimental Protocol

The animals were allocated into six groups. Each group consisted of 12 rats, with 4 per cage. Rats in the Thioacetamide (TAA) group received an intraperitoneal injection of 200 mg/kg TAA daily for 8 weeks. The β-Caryophyllene (BCP) group received a dose of 50 mg/kg by oral gavage daily for 8 weeks. The AM630 group received a daily intraperitoneal injection of 2.5 mg/kg for 8 weeks. The control (CON) group received a vehicle solution of olive oil orally daily for 8 weeks in an equivalent amount to the administered BCP. In the BCP + TAA group, the rats were pretreated with BCP, with an interval of one hour before the TAA injection. The AM630 + BCP + TAA group was injected with AM630 followed by BCP and finally TAA, with a one-hour interval between each.

### 4.4. Tissue Collection

Eight weeks after the experimental treatment, the animals were decapitated, and their blood was collected directly in tubes without the addition of anticoagulants. Surgical dissection was performed to excise the liver, and the liver tissues were washed with phosphate-buffered saline and cut into segments. Some of the liver samples were snap-frozen in liquid nitrogen and stored at −80 °C for later analysis. Others were soaked in 4% formalin, kept for 3 days, transferred to a sucrose solution (30%) at 4 °C for another 3 days and stored at −80 °C for future sectioning.

### 4.5. Tissues Homogenization and Sample Preparation

Liver tissue was mixed in radioimmunoprecipitation (RIPA) assay buffer (Millipore, Burlington, MA, USA) with phosphate and a protease cocktail inhibitor (Thermo Fisher Scientific, Rockford, IL, USA) and homogenized using a tissue homogenizer (Omni International, Kennesaw, GA, USA). The solution was centrifuged at 15,000× *g* at 4 °C for 30 min. The supernatant was pipetted out and stored at −40 °C for biochemical and Western blotting analyses.

### 4.6. Liver Weight Index

Body weight was recorded weekly throughout the treatment duration. Liver weight was recorded after the rats were sacrificed. The liver index was calculated by determining the ratio of liver weight to body weight.

### 4.7. Functional Markers Analysis

On the day of sacrifice, blood was collected for further serum evaluation. The blood samples were centrifuged at 13,000 rpm for 10 min, and the serum was collected. Aspartate aminotransferase (AST) and alanine aminotransferase (ALT) levels were measured using a biochemical kit purchased from MyBioSource (Southern California, San Diego, CA, USA, Cat. No. MBS264975 and MBS269614, respectively).

### 4.8. Malondialdehyde (MDA) Assay

Lipid peroxidation was determined via a spectrophotometric assay using a commercially available kit from Northwest Life Science (Vancouver, WA, USA). The concentration of MDA was measured according to the manufacturer’s protocol. The color absorbance was read at 545 nm by a microplate reader. The concentration was calculated and presented as μM/mL.

### 4.9. Glutathione (GSH) Assay

The concentration of the non-enzymatic antioxidant GSH was estimated using a commercial kit from Sigma-Aldrich (Chemie GmbH, Stein Heim, Germany). Estimation was performed by following the manufacturer’s instructions. The concentration was measured by a microplate reader at 412 nm and presented as μM/mL.

### 4.10. Catalase Assay

Catalase enzyme activity was measured in liver tissue homogenates using an assay kit (Cayman Chemicals Co., Ann Arbor, MI, USA). The enzymatic activity was represented as nmol/min/mL.

### 4.11. Assessment of Pro- and Anti-Inflammatory Cytokines

The levels of the proinflammatory cytokines interleukin-1β (IL-1β), interleukin-6 (IL-6), and tumor Necrosis Factor alpha (TNF-α) and the anti-inflammatory cytokine interleukin-10 (IL-10) were measured using ELISA kits (BioSource International, Camarillo, CA, USA). The cytokine levels are represented as pg/mL.

### 4.12. Western Blotting Assay

The amount of protein in the homogenized liver tissues was estimated using the commercial Pierce TM BCA protein assay kit (Thermo Fisher Scientific, Rockford, IL, USA). Supernatants were mixed with RIPA and Laemmeli buffer (Bio-Rad, Hercules, CA, USA) with 2-mercaptoethanol (Sigma-Aldrich, St. Louis, MO, USA) for sample visualization, heated for 5 min at 95 °C for denaturation, and stored for the subsequent use. The samples were electrophoresed on SDS-PAGE gels and then electro-transferred to PVDF membranes, which were then were immersed in a blocking solution containing bovine serum albumin 5% for 1 h. The membranes were then incubated overnight with the primary antibodies provided in Table 1. Secondary antibodies conjugated to horseradish peroxidase were used to visualize reactive bands with the aid of a chemiluminescence substrate (Thermo Fisher Scientific, Waltham, MA, USA). Quantification was performed using ImageJ software (version 1.54k, National Institutes of Health, Bethesda, MD, USA). GAPDH was used as a loading control for the equalization of total protein expression.

### 4.13. Histological Examination

The fixed liver tissues were embedded in paraffin, and sections were cut at a thickness of 3 μm and collected on slides. The sections were then deparaffinized and stained with Hematoxylin and Eosin. The stained sections were subsequently visualized under a light microscope (Olympus, Hamburg, Germany). Masson’s Trichrome (ab150686; Abcam, Cambridge, UK) and Picrosirius red (ab245887; Abcam, Cambridge, UK) staining was also performed using purchased kits.

### 4.14. Immunohistochemical Staining

The paraffin-embedded sections were stained using avidin–biotin–peroxidase and the 3,3′ diaminobenzidine (DAB) method. The sections underwent an antigen retrieval step of dewaxing and citrate buffer immersion. Primary antibodies (Table 2) were added, and the sections were incubated overnight. Biotinylated secondary anti-mouse and anti-rabbit antibodies were added (1:100) accordingly. An avidin–biotin complex (Vectastain Elite ABC kit, Burlingame, CA, USA) was thereafter added to the slides and left for another 1 h. The sections were then developed with DAB (DAB Kit, Vector Laboratories Inc, Burlingame, CA, USA). Stained sections were mounted using DPX mounting medium and viewed using a light microscope (Olympus, Hamburg, Germany). Staining was quantitatively assessed using ImageJ (version 1.54k) software (National Institutes of Health, Bethesda, MD, USA).

### 4.15. Statistical Analysis

Data was calculated and represented as the mean ± standard error of the mean (SEM). Statistical analysis was performed using one-way analysis of variance (ANOVA) followed by Duncan’s multiple range test (DMRT) on SPSS (version 28.0) statistics software. *p* ≤ 0.05 was considered statistically significant.

## 5. Conclusions

Our findings demonstrate the protective effects of BCP in TAA-induced hepatic fibrosis. They also reveal that the protective mechanisms of BCP are mediated through CB2 receptor activation, which confers antioxidant, anti-inflammatory, and antifibrotic effects in liver fibrosis. BCP was shown to activate the SIRT1 pathway, which mediates the attenuation of oxidative stress and inflammation. Further, BCP treatment was able to reduce fibrotic markers, endothelial transition, and myofibroblast activation. BCP’s pleiotropic effects, negligible toxicity, and dietary availability, in addition to its CB2 receptor-activating property, make it a potential candidate for further development as a nutraceutical and phytopharmaceutical for health benefits and pharmacological management of liver fibrosis. Pharmacokinetic and regulatory toxicology studies are needed to determine its dosage and safety before further studies can be conducted in humans to demonstrate its potential clinical use.

## Figures and Tables

**Table 1:** Primary antibodies used in Western blotting, with their dilution ratio and source.

Antibody	Dilution Ratio	Company and Catalog Number
COL3A1	1:1000	Santa Cruz Biotechnology (Dallas, TA, USA) (sc-271249)
COL1A1	1:1000	Santa Cruz Biotechnology (sc-293182)
SIRT1	1:500	Santa Cruz Biotechnology (sc-74465)
FGF-18	1:1000	Santa Cruz Biotechnology (sc-393471)
HIF-1α	1:500	Santa Cruz Biotechnology (sc-13515)
p-AMPK	1:500	Cell Signaling Technology (Danvers, MA, USA) (2535s)
p-SMAD	1:500	Cell Signaling Technology (8828s)
TGF-β	1:1000	Cell Signaling Technology (3711s)
α-SMA	1:1000	Cell Signaling Technology (14968s)
MMP-2	1:500	Abcam (Waltham, MA, USA) (ab97779)
MMP-9	1:500	Abcam (ab658803)
GAPDH	1:1000	Santa Cruz Biotechnology (sc-365062)

**Table 2:** Primary antibodies used in immunostaining with their dilution ration and source.

Antibody	Dilution Ratio	Company and Catalog Number
Fibronectin	1:50	Santa Cruz Biotechnology (sc-8422)
E-cadherin	1:50	Santa Cruz Biotechnology (sc-52751)
α-SMA	1:50	Cell Signaling Technology (14968s)
Vimentin	1:50	Cell Signaling Technology (5741s)

## Data Availability

Data supporting these findings are available upon request.

## Abbreviations

ALT	Alanine aminotransferase
AMPK	AMP-activated protein kinase
AST	Aspartate aminotransferase
BCP	β-Caryophyllene
CB1, CB2	Cannabinoid receptors 1 and 2
CON	Control
ECS	Endocannabinoid system
FGF-18	Fibroblast Growth Factor
GSH	Glutathione
HIF-1α	Hypoxia inducible factor
IL-1β	Interleukin-1β
IL-10	Interleukin-10
IL-6	Interleukin-6
MDA	Malondialdehyde
MMP	Matrix Metalloproteases
p-SMAD2	Phosphorylated-Caenorhabditis elegans SMA (“small” worm phenotype) and MAD family (“Mothers Against Decapentaplegic”) of genes in Drosophila
SIRT1	Sirtuin 1
TAA	Thioacetamide
TASO	Thioacetamide-S-oxide
TGF-β	Transforming growth factor-beta
TNF-α	Tumor Necrosis Factor alpha
α-SMA	Alpha Smooth Muscle Actin
